# Adaptive laboratory evolution of *Rhodococcus rhodochrous* DSM6263 for chlorophenol degradation under hypersaline condition

**DOI:** 10.1186/s12934-023-02227-7

**Published:** 2023-10-26

**Authors:** Jie Zheng, Zhengzhi Zhang, Juan An, Yubin Xue, Bo Yu

**Affiliations:** 1 CAS Key Laboratory of Microbial Physiological & Metabolic Engineering, State Key Laboratory of Mycology, Institute of Microbiology, Chinese Academy of Sciences, 100101 Beijing, China; 2 University of Chinese Academy of Sciences, 100049 Beijing, China; 3 Linyi Municipal Ecology and Environment Bureau, 276000 Linyi, China

**Keywords:** Chlorophenols, *Rhodococcus rhodochrous*, Bioremediation, Hypersaline, Adaptive laboratory evolution

## Abstract

**Background:**

Normally, a salt amount greater than 3.5% (w/v) is defined as hypersaline. Large amounts of hypersaline wastewater containing organic pollutants need to be treated before it can be discharged into the environment. The most critical aspect of the biological treatment of saline wastewater is the inhibitory/toxic effect exerted on bacterial metabolism by high salt concentrations. Although efforts have been dedicated to improving the performance through the use of salt-tolerant or halophilic bacteria, the diversities of the strains and the range of substrate spectrum remain limited, especially in chlorophenol wastewater treatment.

**Results:**

In this study, a salt-tolerant chlorophenol-degrading strain was generated from *Rhodococcus rhodochrous* DSM6263, an original aniline degrader, by adaptive laboratory evolution. The evolved strain *R. rhodochrous* CP-8 could tolerant 8% NaCl with 4-chlorophenol degradation capacity. The synonymous mutation in phosphodiesterase of strain CP-8 may retard the hydrolysis of cyclic adenosine monophosphate (cAMP), which is a key factor reported in the osmoregulation. The experimentally verified up-regulation of intracellular cAMP level in the evolved strain CP-8 contributes to the improvement of growth phenotype under high osmotic condition. Additionally, a point mutant of the catechol 1,2-dioxygenase, CatA^N211S^, was revealed to show the 1.9-fold increment on activity, which the mechanism was well explained by molecular docking analysis.

**Conclusions:**

This study developed one chlorophenol-degrading strain with extraordinary capacity of salt tolerance, which showed great application potential in hypersaline chlorophenol wastewater treatment. The synonymous mutation in phosphodiesterase resulted in the change of intracellular cAMP concentration and then increase the osmotic tolerance in the evolved strain. The catechol 1,2-dioxygenase mutant with improved activity also facilitated chlorophenol removal since it is the key enzyme in the degradation pathway.

**Supplementary Information:**

The online version contains supplementary material available at 10.1186/s12934-023-02227-7.

## Background

Chlorophenols (CPs) are aromatic components that contain one hydroxyl and at least one chlorine atom substitution on the benzene ring. These products are used in the formulation of bactericides, insecticides, herbicides, fungicides, wood preservatives industry, as well as dyes and pharmaceuticals [1]. CPs and their derivatives are known to possess carcinogenic, mutagenic, and cytotoxic properties, rendering them highly toxic to living organisms [2]. It has been classified as priority pollutants and potential carcinogens to humans by the World Health Organization and the United States Environmental Protection Agency [1]. The wastewater containing CPs is normally accompanied with high salinity, especially in pulp paper industries [3]. During the process of pulp washing, the chlorination process not only chlorinates a large amount of lignin in the pulp, producing CPs, but increases the salinity of the wastewater even approaching 8 ~ 10% (w/v) [4]. The salt amount greater than 3.5% (w/v) is defined as hypersaline [5]. The most critical aspect of the biological treatment of hypersaline wastewater is the inhibitory/toxic effect exerted on microorganisms by high salt concentrations. Under such harsh conditions, it is very difficult to treat by existing strains due to the extreme metabolic stress caused by this hypersaline condition [6]. Thus, the main challenges are to find robust strains to adopt the hypersaline conditions. Consequently, it is necessary to use salt-tolerant and/or halophilic bacteria to conquer the challenge. A previous study showed that salt-tolerant *Halomonas* sp. could improve the phenol biodegradation performance under hypersaline conditions [7]. The removal rate of > 99% on phenol was reported by *Halomonas* in a bioreactor containing 150 g/L salt [5]. Some *Rhodococcus* strains were also reported to degrade aromatic components in salinity condition. *Rhodococcus* sp. W1 and W2 were reported to have phenol degradation ability at 5% NaCl maximumly [8], while studies for degradation of chlorophenol under high salt conditions were limited. A study on saline adaption of activated sludge achieved 91% removal rate to 2-chlorophenol under the condition of 8% NaCl (w/v), but the degrader on strain level was not identified [9]. Another study claimed that the photosynthetic bacteria could degrade 2-chlorophenol with salinity of less than 2.5% NaCl [10]. Degradation of chlorophenols and their derivatives by microbes shared the same metabolism pathway with phenolic compound, which has been well demonstrated. It is generally initiated by the hydroxylation of the functional groups on benzene ring converted by monooxygenase or P450-type-enzymes. Then, the benzene cleavage is mediated by dioxygenases through meta or ortho cleavage pathway. Finally, the ring-cleavage products are channeled into TCA cycle and completely mineralized with release of chloride ion [1]. To date, there is no identified strain capable of degrading chlorophenol under hypersaline conditions (> 3.5%, w/v), which hindered the development of chlorophenol biotreatment technology [7].

Adaptive laboratory evolution (ALE) strategies allow for the engineering of microorganisms by combining genetic variation with the selection of beneficial mutations in an unbiased fashion [11]. ALE has the advantage of letting nonintuitive beneficial mutations occur in many different genes and regulatory regions in parallel. The aimed characters could be easily selected by the growth vigor in special conditions. As a mature and effective technique, ALE has been proven highly effective in the optimization of production strains [12]. For example, the strains of *Lactobacillus plantarum* and *Enterococcus faecium*, which are producers of seaweed biomass, demonstrated a respective increase of 1.29-fold and 1.75-fold in their tolerance to salt, evolved by continuously increasing the gradient of NaCl concentration during cultivation (35, 50, 71, 100, 141, and 200 g/L) [13]. Salt-tolerant *Chlamydomonas* sp. strains that grow well even in the presence of 7% salt were successfully obtained after long-term and continuous cultivation with high salinity [14]. In this study, a salt-tolerant chlorophenol-degrading strain, *R. rhodochrous* CP-8 was successfully created by adaptive laboratory evolution, which could degrade chlorinated phenolic compounds under 8% NaCl condition. The mechanism of salt tolerance was explained by single nucleotide polymorphism (SNP) and functional gene analyses. Thus, this study provided referential information for bioremediation of CPs under hypersaline circumstances.

## Results and discussions

### **High salt-tolerant*R. rhodochrous*CP-8 obtained by adaptive laboratory evolution**

**Fig. 1:**
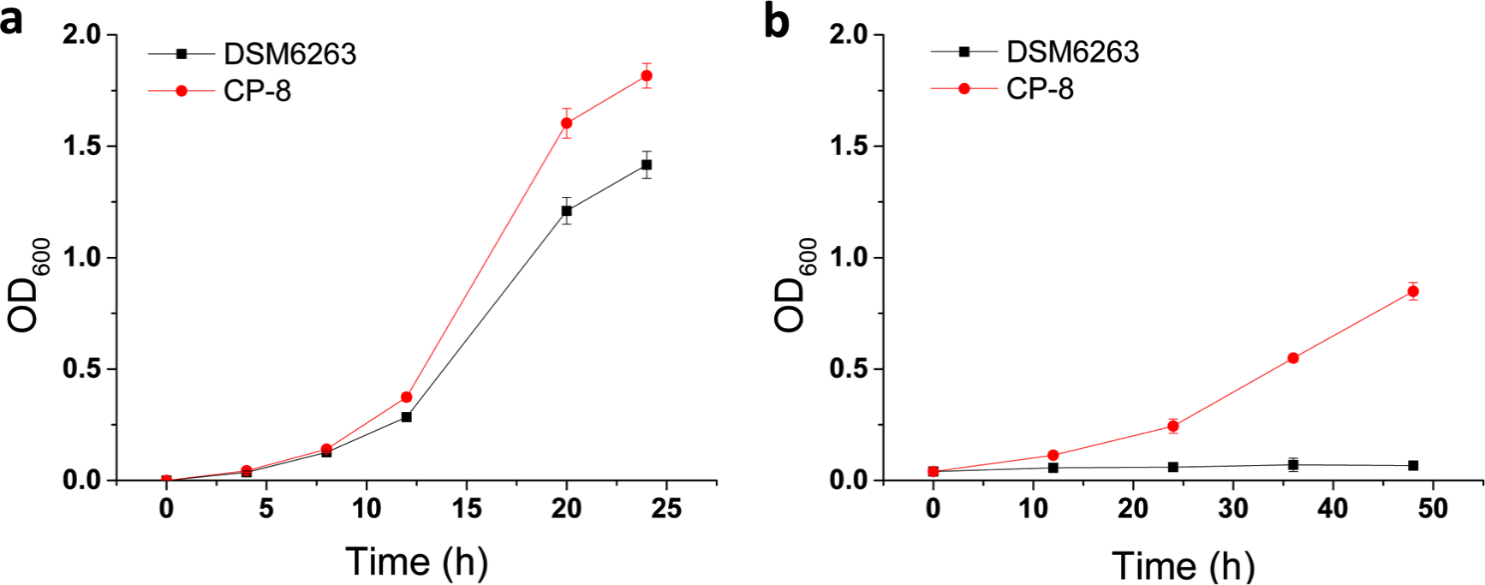
The cell growth of *R. rhodochrous* DSM6263 and CP-8 in M65 medium. **(a)** the growth curve of cells in medium without NaCl addition, and **(b)** the growth curve of cells in medium with 8% NaCl addition. All the experiments were performed in triplicate and data shown are mean ± SD. Some derivations were too small to be shown

**Fig. 2:**
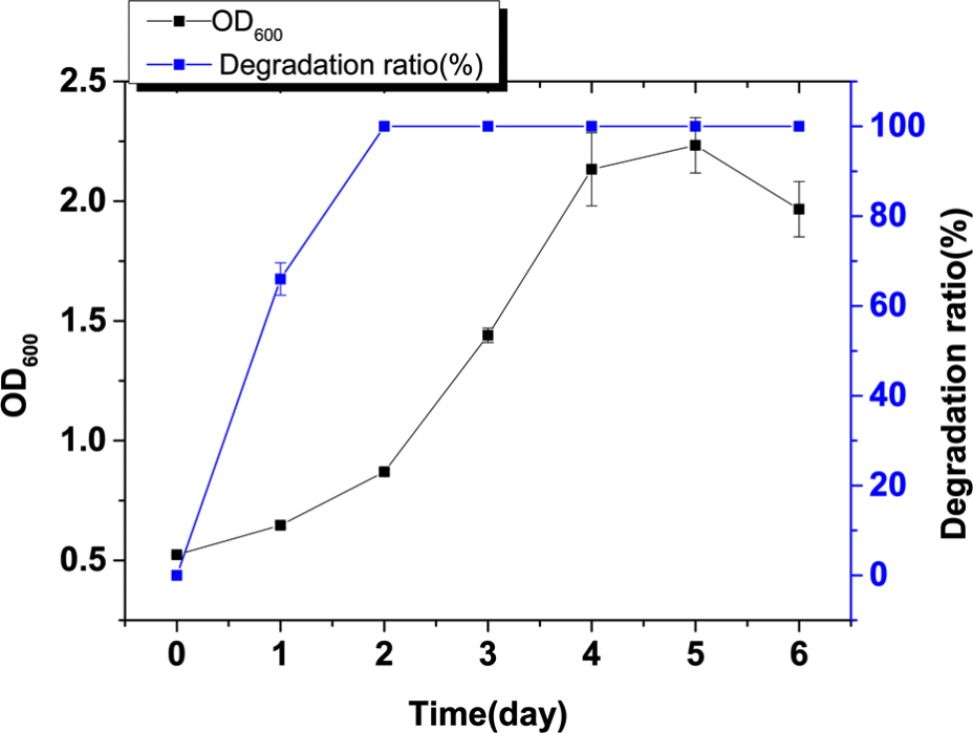
The cell growth and 4-chlorophenol degradation by strain CP-8 under hyperostosis condition with 8% NaCl. All the experiments were performed in triplicate and data shown are mean ± SD. Some derivations were too small to be shown

Following a continuous six-month passage spanning and a total of 63 generations for domestication, a distinct strain named as CP-8 exhibiting heightened salt tolerance of 8% NaCl was successfully isolated. Strain CP-8 grew much better than its parent strain *R. rhodochrous* DSM6263 under both 0% and 8% NaCl conditions (Fig. 1a). There was no obvious growth of DSM6263 in 48 h when 8% NaCl was added in the medium (Fig. 1b). These results demonstrated that the salt tolerance capacity of the strain CP-8 was significantly improved by adaptive laboratory evolution. Then the strain CP-8 was applied to test the 4-chlorophenol degradation ability under high salinity condition. Despite the cell biomass of optical density (OD_600_) reached only 0.7 cultivated under 8% NaCl condition, over 64.7% of 4-chlorophenol was significantly reduced within 1 day of treatment (Fig. 2). 4-Chlorophenol was completely degraded after 2 d, and the biomass accumulated continuously. The cell growth of CP-8 reached stationary phase at Day 4. These results indicated the potential usage of CP-8 in high salinity wastewater treatment.

### Functional gene analysis to demonstrate chlorophenol degradation pathway

Although the parent strain *R. rhodochrous* DSM6263 has been experimentally verified the ability of chlorophenol degradation in our previous study [15], the degradation pathway as well as the responsible genes were not revealed yet. Thus, the whole genome of evolved strain CP-8 was first sequenced. The complete genome of CP-8 consists of a chromosome of 5,098,433 bp, a circular plasmid of 127,620 bp, and a linear plasmid of 124,313 bp. For the KEGG annotation, 2,778 genes were identified in total, and 206 genes were identified as xenobiotics biodegradation and metabolism related genes. Then, 16 genes were mapped into chlorobenzene degradation pathway (KO00361). The degradation pathway of 4-chlorophenol could be divided into upper and downstream pathways. Most of the genes involved in the downstream pathway is widely distributed in microorganisms, even in *E. coli* strain itself. Finally, 5 genes in the upstream pathway of chlorobenzene were selected for functional analysis because of their pivotal roles in CP degradation, including *tfdB* (CP-0GL004302) encoding 2,4-dichlorophenol 6-monooxygenase; *catA* (CP-0GL004530) encoding catechol 1,2-dioxygenase; *dmpB* (CP-0GL004270) encoding catechol 2,3-dioxygenase; *pheA* encoding phenol monooxygenase; *catBC* (CP-0GL004528-CP-0GL4529) encoding muconate cycloisomerase and muconolactone delta-isomerase [16–18].

**Fig. 3:**
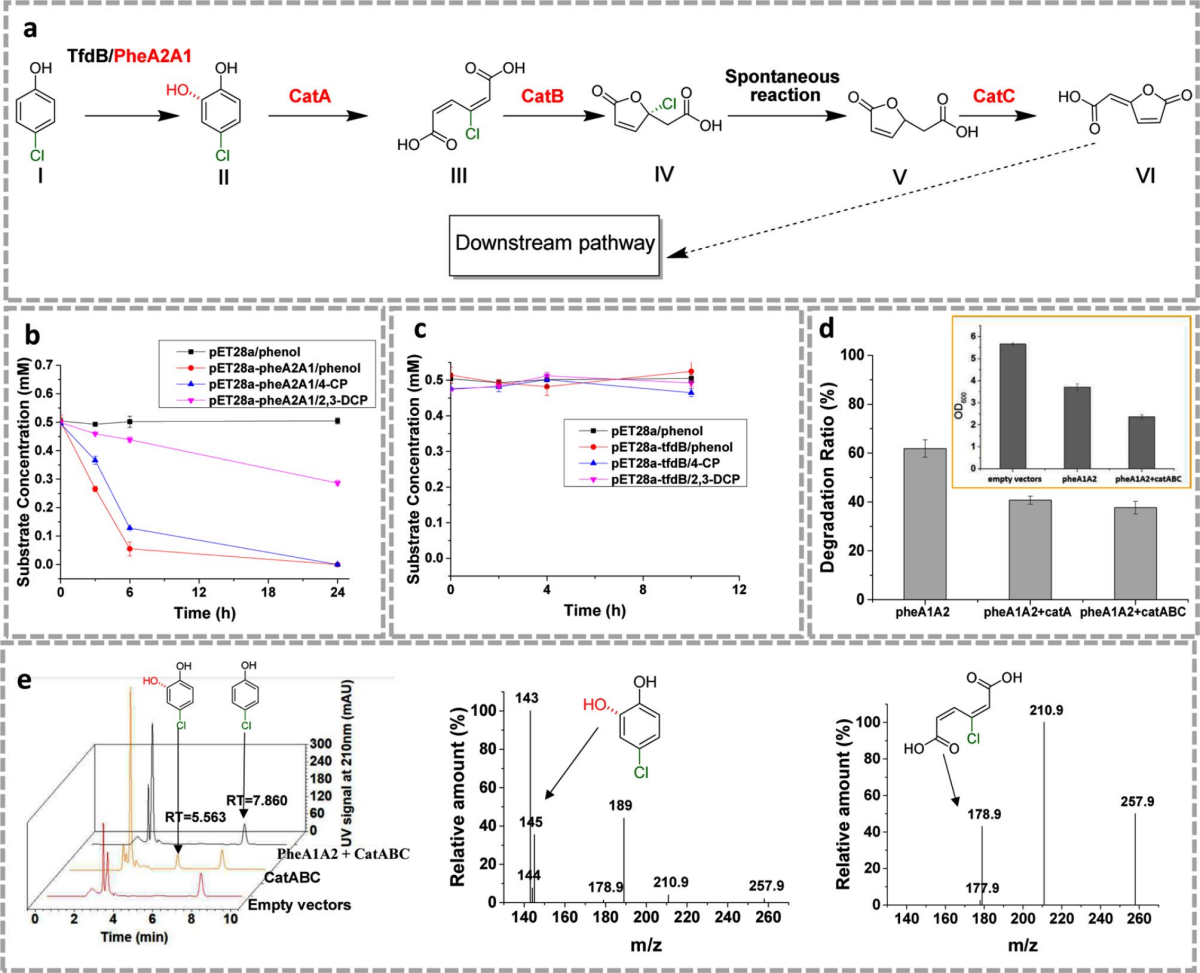
Functional verification of the 4-chlorophenol degradation pathway. **(a)** the proposed 4-chlorophenol degradation pathway. I, 4-chlorophenol; II, 4-chlorocatechol; III, 4-chloromuconic acid; IV, (R)-2-(2-chloro-5-oxo-2,5-dihydrofuran-2-yl) acetic acid; V, 2-(5-oxo-2,5-dihydrofuran-2-yl) acetic acid; VI, (E)-2-(5-oxofuran-2(5 H)-ylidene) acetic acid. **(b)** the degradation of phenol, 4-chlorophenol, 2,3-dichlorophenol by recombinant strain with PheA1A2. **(c)** the degradation of phenol, 4-chlorophenol, 2,3-dichlorophenol by recombinant strain with TfdB. **(d)** the degradation ratio of 4-chlorophenol by different recombinant *E. coli* BL21 strains. pheA1A2, *E. coli* BL21 harboring pET28a-*pheA1A2*; pheA + catA, *E. coli* BL21 harboring pET28a-*pheA1A2* and pYB1s-*catA*; pheA1A2 + catABC, *E. coli* BL21 harboring pET28a-*pheA1A2* and pYB1s-*catABC*; the cell growth of different strains were also indicated; **(e)** HPLC data and mass spectra of the identified metabolic products. All the experiments were performed in triplicate and data shown are mean ± SD. Some derivations were too small to be shown

Based on the genome annotation, the 4-chlorophenol degradation pathway in CP-8 was first proposed in Fig. 3a. The degradation is initiated by ortho hydroxylation to produce 4-chlorocatechol, which might be catalyzed by *tfdB* (2,4-dichlorophenol 6-monooxygenase) or *pheA2A1*(phenol monooxygenase) [19, 20]. The *catA* gene participates in the ortho cleavage of 4-chlorocatechol [16]. The CatBC converts chloro-muconic acid into the downstream mineralization pathway, and chloride ion is spontaneously removed in the process.

In order to further confirm the functions of *tfd*B, *phe*A2A1 and *cat*ABC, these genes were heterologously expressed in *E. coli* BL21(DE3). Besides, DmpB was supposed to perform meta-cleavage activity to chlorocatechol [21]. The annotated DmpB in CP-8 shares 46.8% amino acid sequence homology with DmpB (Uniprot: P31003) from *Geobacillus stearothermophilus*, while the negative 2,3-dioxygenase phenotype has been confirmed by reported experimental evident [22]. In addition, it was reported that the meta cleavage activity of catechol 2,3-dioxygenase was irreversibly poisoned by chlorocatechols [17]. Thus, the gene function of *dmp*B in CP-8 was not tested. TfdB (72.7 kDa), PheA1 (60.1 kDa) and PheA2 (23.9 kDa) were soluble expressed (Figure S1, supplementary file). The ortho hydroxylation enzyme activity to phenol, 4-chlorophenol and 2,3-dichlorocatechol (2,3-DCP) was observed when *phe*A2A1 was expressed in *E. coli* BL21(DE3) (Fig. 3b), while there no enzyme activity was observed by *tfd*B gene (Fig. 3c). This result indicated that the annotation of *tfd*B might be biased. Furthermore, CatABC were co-expressed with PheA2A1 to identify the catechol 1,2-dioxygenase enzyme activity. As shown in Fig. 3d,  the 4-chlorophenol degradation was significantly observed after 6 h incubation. The 4-chlorophenol degradation rate was unexpectedly reduced when CatABC were co-expressed with PheA2A1. One possible explanation might be that too many exogenous genes expressed in one host caused metabolic burden. Thus, the poor physiological status of the strain might cause the decreased degradation ratio. The speculation could be supported by the biomass data of the culture. The final biomass of the strain with CatABC & PheA2A1 co-expression was only 64% of the data of strain with only PheA2A1. Anyway, it should be noted that this experiment focused on the gene function verifications. The degradation rate for 4-chlorophenol in heterologous host was not the point. There was no 4-chlorocatechol accumulation detected by HPLC when CatABC were co-expressed (Fig. 3e), while the accumulation of 4-chlorocatechol was detected in the absence of CatABC. It implied that CatABC does catalyze the degradation process of 4-chlorocatechol. The hydroxylated product of 4-chlorocatechol was identified in LC-MS analysis (Fig. 3e). The degradation product by *E. coli* BL21/PheA2A1 + CatABC were also analyzed by LC-MS and the desired product of 4-chloromuconic acid was confirmed (Fig. 3e). Although the revealed chlorophenol metabolic pathway in CP-8 is consistent with previous reports, the key functional genes involved in *R. rhodochrous* DSM6263 were demonstrated, which provided referential information for engineering the strain in future.

**Fig. 4:**
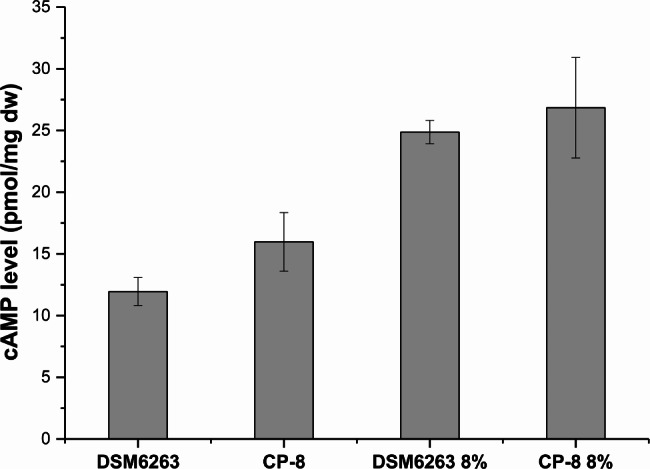
The intracellular concentrations of cAMP in parent strain DSM6263 and evolved strain CP-8 under conditions of 0% and 8% NaCl, respectively. All the experiments were performed in triplicate and data shown are mean ± SD.

### Increased intracellular level of cAMP may be a key factor in osmoregulation

Rhodococci are bacteria that excel in the discipline of overcoming mild, moderate or severe stresses of various kinds. However, the research on stress response is still rare in rhodococci and the molecular mechanism interconnecting these stress responses needs to be investigated. In order to investigate the osmotic tolerance mechanism of evolved strain CP-8, the genome sequence of parent strain *R. rhodochrous* DSM6263 and CP-8 was analyzed in details. SNP analysis revealed 2 interesting point mutations of CP-8 (Table 1). Firstly, ORF GL_002335 mutation G > A causes synonymous mutation of the codon GAG > GAA. ORF GL_002335 was annotated as phosphodiesterase (EC.3.14.17, GOG3540 inorganic ion transport metabolism). This enzyme mediates the hydrolysis of the cAMP and cGMP. Based on the role of cAMP and cGMP in key cellular pathways as secondary messengers [23], the enzyme plays important role in cell signaling [24], including cell inorganic ion transport process [25]. The intracellular level of cAMP is suggested to be a key factor in the osmoregulation [26, 27]. We hypothesized that the mutation may result in the change of intracellular cAMP concentration and then increase the osmotic tolerance in the evolved strain.

**Table 1:** SNP analysis of point mutation genes between strains DSM6263 and CP-8

ORF	Base in DSM6263 genome	Base in CP-8 genome	Codon DSM6263 <->CP-8	AA mutant DSM6263 <->CP-8	Function description
GL_002335	G	A	GAG<->GAA	E<->E	Phosphodiesterase
GL_004530	C	T	AGC<->AAC	N<->S	Catechol 1,2-dioxygenase

Although the site mutation in ORF GL_002335 is synonymous in this study, the point mutation G > A shifted the codon GAG to GAA in strain CP-8. Based on the analysis of codon usage frequency of *R. rhodochrous* from Codon Usage Database (http://www.kazusa.or.jp/codon), the codon usage frequency of the GUG is 0.68 while that of GUU sharply decreased to 0.34. It indicated that the synonymous mutant causes negative influence on phosphodiesterase at the translation level, and decreased the intracellular concentration of the enzyme. That means the cAMP hydrolysis process is decreased, causing the up-regulation of the intracellular cAMP level, which may improve the growth phenotype in high osmotic condition [27]. To prove this speculation, the intracellular concentrations of cAMP in DSM6263 and the evolved strain CP-8 were measured, respectively. As shown in Fig. 4, strain CP-8 exhibited a discernibly elevated cAMP level in contrast to DSM6263, displaying the increment of 1.3-fold in the absence of NaCl. Although the variation became slight under 8% NaCl stressed condition, this phenomenon should further support our speculation that the increased intracellular level of cAMP may be a key factor in osmoregulation, since the cAMP level in parent strain DSM6263 increased obviously as compared to the data in the condition without NaCl stress. There might be other unknown mechanisms that led to the increased intracellular levels of cAMP in cells to cope with 8% salt stress, which further research is needed in the future. Similar phenomenon was also found in other *Rhodococcus* sp. strains. In strain *R. jostii* RHA1, nutrient starvation leads to a lot of transcriptional changes, and the common feature of the most upregulated genes was the presence of a consensus binding sequence for the cAMP-dependent CRP regulator in the promoter region, implying the role of cAMP in the regulation of cell stress resistance [28].

### Catechol 1,2-dioxygenase mutant CatA^N211S^ showed increased enzyme activity

**Fig. 5:**
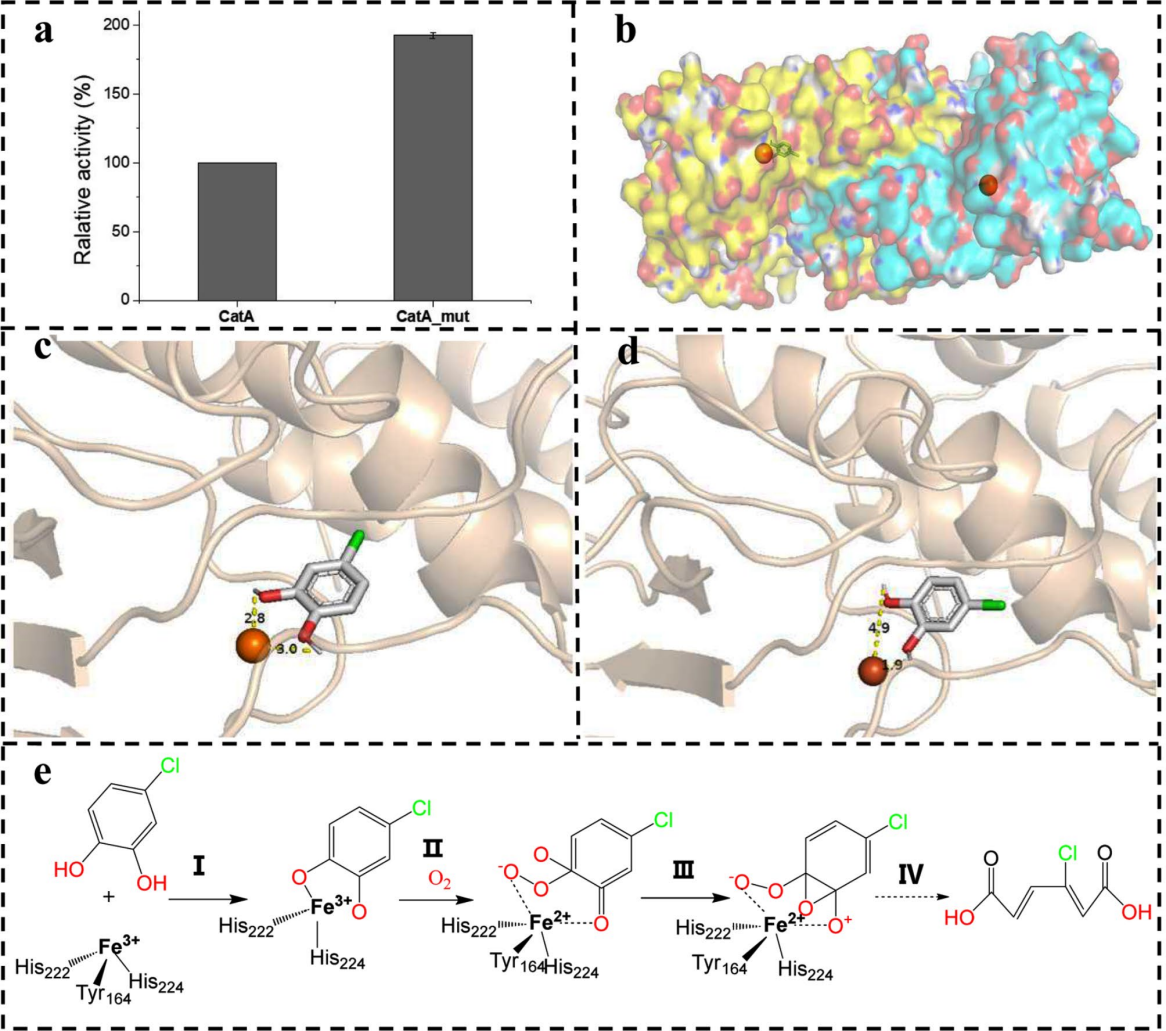
The mechanism of improved enzyme activity of CatA^N211S^ revealed by homologous modeling and molecular docking. **(a)** relative activity to 4-cholocatechol of CatA and CatA^N211S^; **(b)** homodimer structure of CatA calculated by Swiss-Model, Fe(III) is shown as orange ball; **(c)** spatial distance of 4-cholocatechol and Fe(III) in the CatA activity pocket; **(d)** spatial distance of 4-cholocatechol and Fe (III) in CatA^N211S^ activity pocket; **(e)** proposed catalytic mechanism of CatA: I, catechol firstly coordinates with Fe(III) to form a semialdehyde structure; II, oxygen carries out a nucleophilic attack on the unilateral hydroxyl group in an electron transfer process; III, a Criegee 1,2-rearrangement (involving the simultaneous cleavage of the O1-O2 and C3-C4 bonds concerted with formation of a bond between O2 and O3) and a proton transfer process happens; IV leads to muconic acid

Interestingly, another point mutant locates on ORF GL_004530, encoding catechol 1,2-dioxygenase (CatA), which is the key step gene participated in the ortho cleavage of the intermediate chlorocatechol. The mutation C > T causes nonsynonymous mutation of the 211th codon mutant AGC > AAC, resulted in amino acid mutation of N > S (Table 1). It is well known that catechol 2,3-dioxygenases are irreversibly poisoned by chlorocatechols [17], while no mutation site on catechol 2,3-dioxygenase was identified in the evolved strain CP-8. To unravel the mutation on the 4-chlorophenol degradation, the genes of CatA^N211S^ and wild type CatA were expressed in *E. coli* BL21(DE3), respectively. The enzyme activities towards substrate 4-chlorocatechol were compared. CatA^N211S^ showed 1.9-fold relative activity to CatA by the whole-cell catalyst (Fig. 5a). The kinetic parameters of both CatA and CatA^N211S^ were undergone characterization (Table 2). The K_cat_ value of CatA^N211S^ surpasses that of CatA by a fold of 1.30, alongside an associated elevation in K_cat_/K_m_ by 1.16-fold. Despite the advantageous elevation in the turnover number, it is noteworthy that the increase in the K_m_ value bears a contrary influence on substrate binding to the enzyme. Nevertheless, the pronounced augmentation in the turnover number remains pivotal in driving the observed improvement. Given that CatA from *R. rhodochrous* DSM6263 exhibits the most substantial protein homology (61%) with CatA of *R. opacus* 1CP (3i51.1.A) in Protein Data Bank (PDB), we employed the crystal structure of CatA from *R. opacus* 1CP as a template for homologous modeling and molecular docking. The outcomes of these analyses revealed the formation of homodimers for both CatA and CatA^N211S^. Furthermore, it was observed that within each subunit, the active site hosts 4-chlorocatechol (Fig. 5b).

**Table 2:** The parameters of CatA and mutant CatA^N211S^

	CatA^N211S^	CatA
K_m_ (µM)	504.33	451.61
K_cat_ (min^− 1^)	5.98	4.61
K_cat/Km_ (µM^− 1^ min^− 1^)	0.012	0.010
_Δ_G (kcal mol^− 1^)	-5.1	-6.1

To demonstrate the mechanism of enzyme activity increasement, the modeling structure was analyzed in details. In the active site of each subunit, Fe(III) is coordinated to the residues His222, His224 and Tyr164, which have been confirmed as conserved motif of endodiol dioxygenase in previous study [29]. The point mutant N211S did not change the coordinate way of Fe(III). In contrast, it altered the way of binding of the substrate to the iron ion and the orientation of the aromatic ring in the complex. Residues Pro107 and Tyr198 bind the aromatic ring, and residues Ile104, Arg219, Ala252, Val81 bind the chlorine in 4-position for the wild type CatA while residues Pro107, Arg219, Tyr198 and Ala252 of CatA^N211S^ bind the aromatic ring. Leu77, Ile200, Val81, and Tyr198 bind the chlorine in 4-position. These differences lead to a changed distance between 4-chlorophenol hydroxyl group and the iron atom (Fig. 5c-d). Wild type CatA represents 3.0 Å distant to hydroxyl group in position 1 and 2.8 Å distant to hydroxyl group in position 2, while the distance in CatA^N211S^ represents 1.9 Å and 4.9 Å respectively. Ferric ion in the active cave of dioxygenase mediates electron transport during oxygenation process [29–31]. A widely accepted hypothesis of the catalytic mechanism of dioxygenase proposed that catechol coordinates with Fe(III) firstly to form a semialdehyde structure [32]. Then oxygen carries out a nucleophilic attack on the unilateral hydroxyl group in an electron transfer process mediated by iron atom to form a peroxide bridge (Fig. 5e) [33]. Moreover, a Criegee 1,2-rearrangement (involving the simultaneous cleavage of the O1-O2 and C3-C4 bonds concerted with formation of a bond between O2 and O3) and a proton transfer process happens, which leads to muconic acid formation [29, 30]. Transient and steady-state kinetic studies reveal that O_2_-dependent conversion of the chromogenic binary complex (nucleophilic attack on the unilateral hydroxyl group) is rate-limiting process for the overall reaction [34]. In a study of biomimetic chemical catalysts, a series of 3,5-di-*tert*-butylcatechol catalytic models with ferric-oxygen bridging bond were established, a negative correlation between the oxidation potential and the chemical reaction rate constants were demonstrated through measurement and linear-fitness of the enzymatic parameter [33]. Inspired by these evidences, we claimed the proposed mechanism of the catalysis activity improvement. The hydroxyl group in position 1 represents more close space distance to Fe (III). The closer spatial distance causes lower oxidation potential between the hydroxyl group and the iron atom, indicating a higher turnover number. In a computational simulation on the intradiol cleaving enzyme, researchers highlighted the significance of the in-plane geometry of the peroxo intermediate. According to their results, the peroxo species undergoes a geometric transition that allows an X-OH moiety (H_2_O or Tyr447) to occupy the axial position [35]. These results supported our hypothesis that the unilateral hydroxyl group is involved in electron transport. Moreover, within the context of molecular docking investigations, an intriguing observation emerged in relation to the Gibbs free energy associated with the CatA^N211S^-substrate complex (Table 2). The discernibly higher Gibbs free energy signifies a relatively weakened substrate binding affinity. These pieces of evidence collectively elucidated the underlying basis for the amplified K_m_ value of CatA^N211S^. The identified point mutant also provided referential information for further improving the catechol 1,2-dioxygenase activity by rational design, which the enzyme initiates the key step in aromatic compound biodegradation.

## Conclusion

One chlorophenol-degrading strain *R. rhodochrous* CP-8 with extraordinary capacity to tolerate high salinity condition (8% NaCl) was successfully generated by adaptive laboratory evolution in this study. It is the first strain reported to degrade CPs under hypersaline condition. The increase of intracellular cAMP level might be the key factor of improved salt tolerance in the evolved strain CP-8. Additionally, the mutant of catechol 1,2-dioxygenase, CatA^N211S^, in strain CP-8 generated 1.9-fold activity improvement, which should also facilitate CPs degradation. The mechanism of activity increment of CatA^N211S^ was explained by the hypothesis on nucleophilic attack of ferroins on hydroxyl group. In conclusion, this study provides some knowledge on biodegradation of chlorophenols under hypersaline condition. However, more in-deep study on mechanism of salt tolerance are needed to be further explored. Additionally, application of the evolved strain CP-8 to treat real pulp wastewater, of which a complex system contains multiple pollutants and inhibitory factors, is under investigation in our team.

## Materials and methods

### Adaptive laboratory evolution to increase salt tolerance

*Rhodococcus rhodochrous* DSM6263 was purchased from Deutsche Sammlung von Mikroorganismen und Zellkulturen GmbH (DSMZ). The strain DSM6263 was initially cultured in 1/10 LB medium (1 g/L Tryptone, 0.5 g/L YE) at 30℃, 220 rpm for 3 days. It was sub-cultured in 1/10 LB medium with a gradually increased NaCl concentration from 0 to 8% (w/v). Besides, 200 mg/L phenol, used as the model substrate, was added for the enrichment of the degradation phenotype in every generation. The salinity was set at 3% to initiate the evolutionary adaptation process. Once the cell growth reached a stable state, an increment of the salt concentration by 0.5% for the next round of domestication was applied until to the NaCl concentration of 8% at the end of adaptation process. The passage was carried out a total of 63 times in 6 months. Singe clone was isolated on LB plate containing 8% NaCl (w/v). One strain, named as *R. rhodochrous* CP-8, was confirmed with much improved salt-tolerance and phenol biodegradation ability.

### Degradation of 4-chlorophenol by strain CP-8 in hyperostosis condition

CP-8 cells were inoculated in 250 mL flask containing 100 mL M65 medium with 8% NaCl and incubated at 30 °C and 220 rpm for 3 days for seed preparation. The M65 broth contained 4 g/L yeast extract, 4 g/L glucose and 10 g/L malt extract. The seed culture was then inoculated into fresh M65 medium with 8% NaCl and 20 mg/L 4-chlorophenol and incubated at 30 °C for 7 days. The cell optical density (OD_600_) and the residual concentration of 4-chlorophenol were measured every day.

### Functional gene analysis for chlorophenol degradation

The annotated genes in the genome of *R. rhodochrous* DSM6263 were heterologously expressed in *E. coli*. Gene *tfdB* encoding 2,4-dichlorophenol 6-monooxygenase, and gene *pheA* encoding phenol monooxygenase were cloned to vector pET28a, respectively. Gene *catA* encoding catechol 1,2-dioxygenase and the gene cluster *catABC* encoding catechol 1,2-dioxygenase, muconate cycloisomerase and muconolactone delta-isomerase were cloned into vector pYB1s [36]. The recombinant plasmids were transformed into *E. coli* BL21(DE3) cell individually or together for heterologous expression. The recombinant *E. coli* strain harboring plasmids was cultivated in 100 ml of LB medium at 37℃. When the OD_600_ reached a value of 0.6–0.8, the culture was induced by adding 0.5 mM IPTG for gene expression with plasmid pET-28a derivatives, and 0.2% arabinose for pYB1s derivatives at 16℃ for 20 h to induce the gene expressions. The culture was harvested by centrifugation and resuspended in the 100 mM PBS to a final OD_600_ = 15. Initially, 0.5 mM 4-chlorophenol was added. For testing the activity of catechol 1,2-dioxygenase (CatA), the substrate 4-chlorocatechol was prepared by whole cells of *E. coli* BL21(DE3)/pET28a-*pheA2A1*. In order to rule out the possible effect of photodegradation, *E. coli* BL21(DE3) cell with empty plasmid was set as control. The enzyme activity increasement of the mutant CatA^N211S^vs. the wild-type was defined as the following equation: $Relative activity=\frac{{C}_{0}^{mut}-{C}_{1}^{mut}-\varDelta {C}_{control}}{{C}_{0}^{wt}-{C}_{1}^{wt}-\varDelta {C}_{control}}\times 100\%$. All the information on the strains and plasmids used in this study is listed in Table 3.

**Table 3:** Strains and plasmids used in this study

Strains	Description	Reference or source
*Rhodococcus rhodochrous* DSM6263	Wild type strains	Purchased from DSMZ
*E. coli* BL21(DE3) *E. coli* MG1655	Used for heterologous expression Used for heterologous expression	TransGen Biotech, China Laboratory stock
*R. rhodochrous* CP-8	Generated from *R. rhodochrous* DSM6263	This study
**Plasmids**		
pET-28a	Used for heterologous expression	Laboratory stock
pYB1s	Used for heterologous expression	[34]
pET28a-*pheA1A2*	pET-28a harboring gene *pheA1A2*	This study
pET28a-*tfdB*	pET-28a harboring gene *tfdB*	This study
pYB1s-*catABC*	pYB1s harboring gene *catABC*	This study
pYB1s-*catA*	pYB1s harboring gene *catA*	This study
pYB1s-*catA*^N211S^	pYB1s harboring gene *catA*^N211S^	This study

### Genome and SNP analysis between strains DSM6263 and CP-8

Whole genome sequencing (WGS) was performed on the Illumina MiSeq and Pacbio Sequel platforms by Shenzhen BGI Co. LTD, China. Genomes were assembled by Canu [37]. Glimmer 3.02 [38] was used to predict the CDS. Non-coding RNA was predicted by tRNAscan [39] and RNAmmer [40]. CDS was annotated base on GO, KEGG, Swiss-Prot and NR respectively. For SNP analysis, clean data of CP-8 was mapped to reference genome DSM6263. Nucleotide frequency was counted by silicon program.

### Determination of in vivo cAMP levels.

DSM6263 and CP-8 was initially cultured in M65 medium without NaCl for 12 h, Subsequently, 8% NaCl was introduced into the medium, and the cultures were further incubated for an additional 6 h. After centrifugation and bacterial pellet collection, rapid freezing was achieved using liquid nitrogen. The bacteria were then disrupted utilizing the liquid nitrogen grinding approach. A solution of 10% methanol was subsequently employed to resuspend the cell homogenate. cAMP was assayed using high-performance liquid chromatography (HPLC) performed with an Agilent 1260 infinity instrument equipped with a reverse-phase poroshell 120 EC-C18 column (4.6 × 150 mm, 2.7 μm, Agilent). The mobile phase was a mixture of methanol and 100mM KH_2_PO_4_ (10:90 [vol/vol]) at a flow rate of 1 mL/min. The injection volumes for all samples were 5 µl, respectively and monitored at 254 nm with a variable-wavelength detector.

### Homologous modeling and molecular docking

Homologous modeling of CatA and CatA^N211S^ structure follows the standardized process of Swiss-Model. Molecular docking was performed by program AUTODOCK 4.2 [41]. Protein and molecular structure were visualized by PyMOL2.

### Characterization of the kinetic parameters of catechol 1,2 dioxygenase

The kinetic parameters were determined by employing a substrate concentration from 1 mM to 1 µM at 30℃ in triplicate (enzyme concentration 5 µM). The data were fitted to the Michaelis-Menten model by a non-linear least-squares fitting program The reactions was assayed by spectrophotometric measurement of absorbance increase at 262 nm corresponding to the conversion of chlorocatechol into *cis-cis* cholromuconic acid.

### Analytical methods

Cell biomass was measured in terms of optical density at 600 nm (OD_600_). The residual phenol and 4-chlorophenol contents were determined using high-performance liquid chromatography (HPLC) performed with an Agilent 1260 infinity instrument equipped with a reverse-phase poroshell 120 EC-C18 column (4.6 × 150 mm, 2.7 μm, Agilent). The mobile phase was a mixture of methanol and MillQ water (60:40 [vol/vol]) at a flow rate of 0.8 mL/min. The injection volumes for all samples were 5 µl and monitored at 210 nm with a variable-wavelength detector. LC-MS analysis was performed with a SHIMADZU LCMS-2020 instrument. Mass spectrometry was performed in ESI positive- and negative-ion modes with a scan range 50–500 *m/z*. The ESI-MS interface parameters were set as follows: drying gas (nitrogen) flow rate, 1.5 L/min; capillary column temperature, 350℃; spray voltage, 4.5 kV.

### Electronic supplementary material

Below is the link to the electronic supplementary material.

Supplementary Material 1

## Data Availability

The authors confirm that the data supporting the findings of this study are available within the article and its supplementary materials.
