# Supplementary material for: Adaptive laboratory evolution of Rhodococcus rhodochrous DSM6263 for chlorophenol degradation under hypersaline condition
Source: Microb Cell Fact. 2023 Oct 26;22:220. doi: 10.1186/s12934-023-02227-7 (PMC10601206; doi:10.1186/s12934-023-02227-7)
Supplement: Supplementary file 1 — Supplementary Material 1 [file 12934_2023_2227_MOESM1_ESM.docx]

Supplementary information

**Adaptive laboratory evolution of** ***Rhodococcus* *rhodochrous*** **DSM6263 for chlorophenol degradation** **under** **hypersaline condition**

Jie Zheng^1,2^, Zhengzhi Zhang^3^, Juan An^1,2^, Yubin Xue^1^, Bo Yu^1*^

*^1^ CAS Key Laboratory of Microbial Physiological & Metabolic Engineering, State Key Laboratory of Mycology, Institute of Microbiology, Chinese Academy of Sciences, Beijing 100101, China*

*^2^ University of Chinese Academy of Sciences, Beijing 100049, China*

*^3^ Linyi Municipal Ecology and Environment Bureau, Linyi 276000, China*

For correspondence:

^*^E-mail: yub@im.ac.cn

Tel./Fax +86-10-64806132

**Table S1** The 16 genes mapped to chlorobenzene degradation pathway

| **GeneID** | **KoNumber** | **Identity** | **Ko_name** | **Ko_defi** | **Ko_EC** |
| --- | --- | --- | --- | --- | --- |
| CP-0GL001029 | ko00361 | 99.7 | None | maleylacetate reductase | 1.3.1.32 |
| CP-0GL002467 | ko00361 | 56.2 | dehH | haloacetate dehalogenase | 3.8.1.3 |
| CP-0GL002575 | ko00361 | 82 | chqB | hydroxyquinol 1,2-dioxygenase | 1.13.11.37 |
| CP-0GL002576 | ko00361 | 82.6 | None | maleylacetate reductase | 1.3.1.32 |
| CP-0GL003860 | ko00361 | 55.3 | None | 2-haloacid dehalogenase | 3.8.1.2 |
| CP-0GL003962 | ko00361 | 98.7 | None | hydroxyquinol 1,2-dioxygenase | 1.13.11.37 |
| CP-0GL003963 | ko00361 | 75 | None | maleylacetate reductase | 1.3.1.32 |
| CP-0GL004134 | ko00361 | 100 | None | carboxymethylenebutenolidase | 3.1.1.45 |
| CP-0GL004270 | ko00361 | 99.7 | dmpB | catechol 2,3-dioxygenase | 1.13.11.2 |
| CP-0GL004302 | ko00361 | 97.8 | tfdB | 2,4-dichlorophenol 6-monooxygenase | 1.14.13.20 |
| CP-0GL004348 | ko00361 | 45.7 | pcpD | tetrachlorobenzoquinone reductase | 1.1.1.404 |
| CP-0GL004520 | ko00361 | 40.1 | pcpD | tetrachlorobenzoquinone reductase | 1.1.1.404 |
| CP-0GL004529 | ko00361 | 90.7 | catC | Muconolactone Delta-isomerase | 3.1.1.45 |
| CP-0GL004529 | ko00361 | 88.5 | catB | muconate cycloisomerase | 5.5.1.1 |
| CP-0GL004530 | ko00361 | 100 | catA | catechol 1,2-dioxygenase | 1.13.11.1 |
| CP-0GL005107 | ko00361 | 88.5 | None | None | 5.5.1.1 |

**Figure S1** The protein expression of TfdB and pheA1A2 by SDS-PAGE analysis


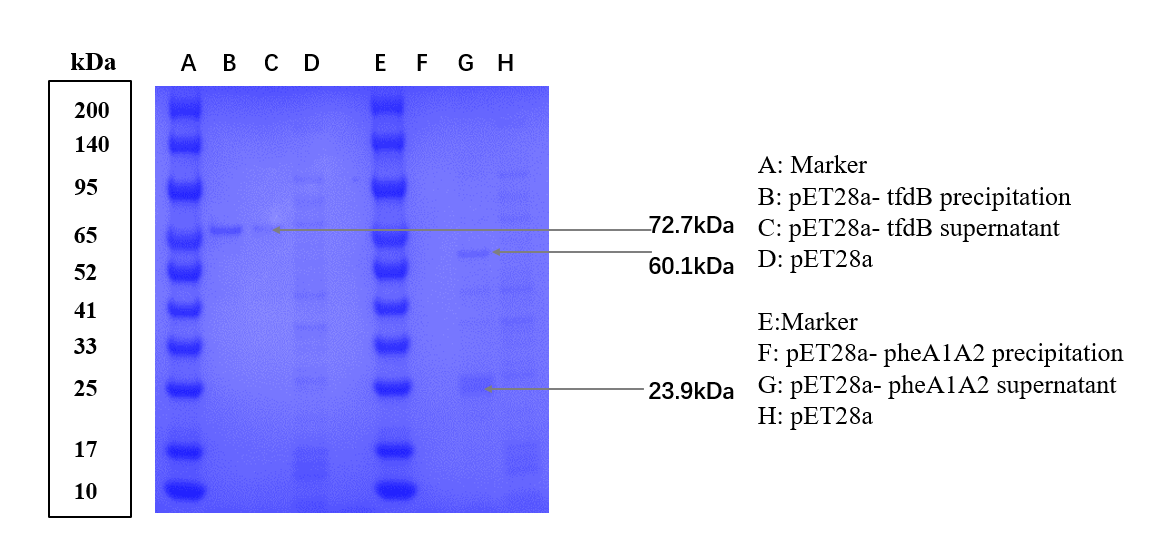


A, Marker; B, pET28a-*tfdB* precipitation; C, pET28a-*tfdB* supernatant;

D, pET28a empty vector; E, Marker; F, pET28a-*pheA1A2* precipitation;

G, pET28a-*pheA1A2* supernatant; H, pET28a empty vector.
